# Characterization of biosurfactants’ micelles formation using fluorescence measurements: sodium taurocholate as case of study

**DOI:** 10.5599/admet.2322

**Published:** 2024-07-16

**Authors:** Susana Amézqueta, Elisabet Fuguet, Rubén Cabañas, Clara Ràfols

**Affiliations:** 1 Departament d'Enginyeria Química i Química Analítica, Universitat de Barcelona, Martí i Franquès 1-11, 08028, Barcelona, Spain; 2 Institute of Biomedicine of the University of Barcelona (IBUB), Universitat de Barcelona, Martí i Franquès 1-11, 08028, Barcelona, Spain; 3 Serra Húnter Programme, Generalitat de Catalunya, Barcelona, Spain

**Keywords:** Bile salts, critical micelle concentration, fluorescence spectrophotometry, biorelevant media

## Abstract

**Background and purpose:**

The evaluation of micellization parameters of surfactants that aggregate gradually, such as bile salts, is not trivial. In this work, different probes and data treatment models are tested to set up an analytical method based on fluorescence measurements to determine the critical micelle concentration (CMC) and micellization range ((*C*) of biosurfactants. Sodium taurocholate (NaTc) is used as example.

**Experimental approach:**

The fluorescence intensity of five fluorophores has been monitored upon the addition of a concentrated NaTc solution in two different media: water and a biorelevant buffer (maleic buffer pH 6.5, *I* = 120 mM). Four different data treatment methods have been tested.

**Key results:**

The micellization process can be evaluated satisfactorily using fluorescent probes such as propranolol and tetracaine, and also monitoring directly the intrinsic fluorescence of NaTc. However, the results obtained with nonpolar probes (pyrene and naphthalene) are more complex to evaluate due to the presence of confluent processes. Although the four models tested for the data treatment are commonly used for this purpose, Carpena’s method is the most appropriate as it provides the most accurate CMC and Δ*C* values. The micellization process is faster in a biorelevant buffer than in water.

**Conclusion:**

The study of the micellization of bile salts is not an evident process. After the selection of adequate probes and data treatment methods, the CMC values for NaTC in water and maleic buffer reveal that the biorelevant conditions favour micellization, which in turn may allow faster solubilization of ingested compounds.

## Introduction

**Figure 1.**
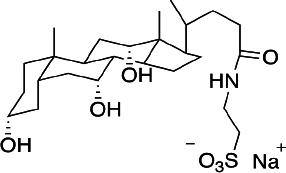
Sodium taurocholate structure

Sodium taurocholate (NaTc) (Figure 1) is a salt secreted by bile and present in gastrointestinal fluids. This amphiphilic molecule can form micelles and, therefore, has very interesting physicochemical characteristics at a biological level. The micelles can house substances with low solubility in aqueous media, such as most drugs in common use administered orally. Incorporating into the structure improves the solubilization of bioactive substances in gastrointestinal fluids, favoring their absorption and transport to the site/s of action.

Conventional surfactants such as sodium dodecyl sulphate (SDS) have a hydrophobic tail and a hydrophilic head and at low concentrations (~10 mM), form micelles with spherical shape [1]. In the case of NaTc and other biliary salts, the molecule has a hydrophilic part made up of hydroxyl groups and a sulfonate group and a nonpolar part made up of methyl groups. Due to their structure, bile salt micelles have smaller aggregation numbers, higher charge density and higher polydispersity than conventional surfactants [2]. Notwithstanding, there exists still an open debate about the exact aggregation process and the micelles’ structure [2,3]. In this sense, one of the most widely accepted is Small’s model [4,5], which suggests that the bile salt hydrocarbon parts of 2-9 monomers are orientated inwards and the hydrophilic groups outwards, forming a globular micelle. Further studies have indicated that the aggregation of bile salts would occur in a stepwise manner [6,7]. The most recent studies performed using NMR [4] have shown that the bile salt molecules aggregate with the convex side of molecules aligning through the hydrophobic interaction of two methyl groups. In the specific case of NaTc, they observed that the aggregates are small and the interactions between molecules are very complex [4]. One of the main parameters evaluated when studying micellization is the critical micelle concentration (CMC). Although in the past, the use of the term “noncritical multimer concentration” was suggested instead of CMC for those associations that occur gradually [8], the most extended term is still CMC and will be used in the present work. Surfactants CMC value can be influenced by the temperature, the overall surfactant concentration, the presence of other components in the solution (such as other surfactants), the ion strength and the pH [9] and, therefore, the micellization and the drugs’ solubilization ability of NaTc and other bile salts will probably be different in water than in intestinal fluids.

Micellization can be evaluated by monitoring one physicochemical property that undergoes a variation when monomers aggregate, such as conductivity [10], osmotic concentration [10], fluorescence [7,11,12], nuclear magnetic resonance [13] and ultrasound velocity [14]. Here, fluorescence was selected due to its wide availability and high sensitivity using low amounts of reagents. Usually, fluorescence methods for CMC determination monitor a probe upon successive additions of the surfactant. The probe fluorescence intensity *vs.* surfactant concentration linear trend changes when micelles are formed and the breakpoint of the two linear trends is set as the CMC value.

Nonetheless, the type of probe used for monitoring the fluorescence intensity plays an important role in the determination. McGown *et al.* tested using different probes for their studies on NaTc micellization. In a first study, they evaluated the use of fluorescein isothiocyanate, perylene, benzo[κ]fluoranthene, chrysene and tetracene [7]. In the second one, they used pyrene, benzo[e]pyrene, benzo[g,h,i]perylene, coronene and ovalene [11]. The authors compared the spectra of the probes in the presence of the surfactant with the spectra of the probes solved in water and in an organic solvent, and also the influence of NaTc concentration on the relative fluorescence intensity of the probes. They concluded that when the probes are solubilized into the micelle and interact with the aggregates, their environment becomes relatively hydrophobic, and the fluorescence intensity of the nonpolar-favoured peaks increases. In the first study, they indicated that the formation of primary micelles (presumably composed of tetramers [7] or pentamers [15]) takes place gradually, approximately in the range of 8-12 mM of NaTc at 25 °C. In the second study, they showed how probes with different sizes can detect the formation of the primary micelles at about 8-12 mM of NaTc at 20 °C and other aggregates (from dimers to bigger structures) depending on the selected probe. Notwithstanding, these two initial studies were semiquantitative.

The definition of the CMC and micellization ranges is not obvious. Once the influence of the surfactant concentration on the probe fluorescence has been monitored, different calculation methods exist to set the CMC value and the micellization range. Some of them are based on the same principles as those used for conductometry methods [16], but they have different sensitivity and accuracy depending on the type of surfactant. Others use pyrene as the probe and model the pyrene 1:3 ratio data using a Boltzmann-type sigmoid [17].

**Figure 2.**
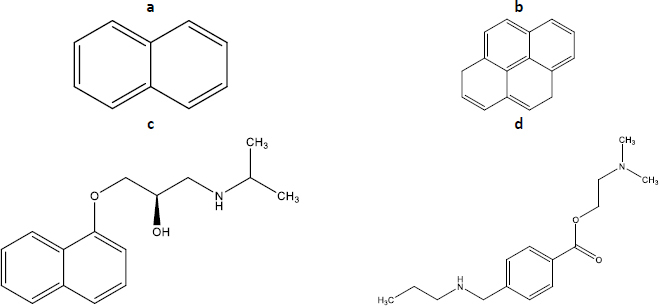
Structure of: a - naphthalene, b - pyrene, c - propranolol, d - tetracaine.

The present work is focused on evaluating and comparing sodium taurocholate micellization parameters (CMC and micellization range) in two different media, water and a relevant buffer. The biorelevant buffer selected (maleic buffer, pH 6.5, *I* = 120 mM) mimics both the pH and the ion strength of intestine fluids in the fasted state [18]. The study will be performed by fluorescence spectroscopy using four different probes (pyrene, naphthalene, propranolol and tetracaine, Figure 2). Also, the direct monitoring of NaTc, which presents intrinsic fluorescence, will be considered. The adequation of the different available calculation methods will also be discussed for the case of NaTc.

## Experimental

### Reagents

Sodium dodecyl sulphate (SDS) ≥99 %, sodium taurocholate ≥95 %, pyrene ≥99 %, naphthalene ≥95 %, propranolol hydrochloride ≥99 %, tetracaine ≥99 % and dimethyl sulfoxide (DMSO) ≥99 % were from Merck (Darmstadt, Germany). All compounds were dissolved in water previously purified using the Mili-Q™ plus System with a resistivity of 18,2 MΩ cm from Millipore (Billerica, MA, USA) or in the corresponding buffer. To prepare the buffers, sodium chloride ≥ 99.5% from Fisher Scientific (Hampton, NH, USA), maleic acid ≥99.5 % from Carlo Elba (Milano, Italy), and sodium hydroxide ≥98 % from Merck (Darmstadt, Germany) were used. Buffers were prepared according to the suggested procedure to mimic the ion strength and pH of intestinal fluids [18].

### Equipment

Fluorescence measurements were performed with a Cary Eclipse fluorescence spectrophotometer (Agilent Technologies, Santa Clara, MA, USA). The buffers were prepared using a GLP 22 5014 combination electrode pH meter from Crison (Barcelona, Spain).

### Methods

First, a stock solution of different probes was prepared in the selected medium (water or buffer) or DMSO for naphthalene and pyrene due to their limited solubility in water. Next, a working solution was prepared in the desired medium (~60 μM for propranolol, 30 μM for tetracaine and, 2 μM for naphthalene and 0.03 μM for pyrene). In the case of naphthalene and pyrene, the amount of DMSO in the final solution is very small and does not have an influence in further experiments (data not shown, verified by ITC). Also, a solution of the surfactant (~300 mM) was prepared in the desired medium.

Three mL of the probes working solutions or the selected medium were placed in a cuvette. Next, several additions of 10-15 1L of NaTc were done within 3 min intervals, covering the premicellar and the postmicellar regions (up to 10-18 mM). The temperature of the experiment was set at 25 °C. Fluorescence measurements after each addition were performed using a 1 cm path length quartz QS cuvette (Hellma Analytics, Jena, Germany), at λ_ex_ =310 nm, λ_em_ = 364 nm for NaTc; λ_ex_ =310 nm, λ_em_ = 349 nm for propranolol; λ_ex_ =310 nm, λ_em_ = 372 nm for tetracaine; and λ_ex_ =280 nm, λ_em_ = 320 nm for naphthalene. In the case of pyrene, the wavelengths monitored were at λ_ex_ =335 nm, λ_em_ = 384 nm (the so-called I_3_ vibronic band) [17]. The slit width of the monochromators was set at 10 nm and the scan speed was set at 600 nm/min. The experiments were done, at least, in triplicate.

### Calculations

First, it must be noted that maleic buffer and NaTc are fluorescent; hence, when applicable, their contribution has to be subtracted from the total measured fluorescence to obtain the signal of the fluorophore of interest.

The fluorescence of NaTc and the probes that are soluble in their micelles have a linear behaviour in the premicellar and the postmicellar regions but present a different slope. The same phenomenon is observed in the conductometric determination of the CMC. Goronja *et al*. [16] applied four different calculation methods to obtain the CMC from conductivity measurements. The same methods will be applied in the present study to evaluate the fluorescence data:

- Conventional or graphical method (method 1). The plot of fluorescence intensity (*F*) *vs.* NaTc concentration (*C*) shows two different linear segments. CMC is set as the intersection of the two straight lines presumably corresponding to the premicellar and the postmicellar regions.
- First derivative method (method 2). The derivative ∂*F*/∂*C vs. C* is sigmoidal and can be modelled using the Boltzmann equation (Eq. 1):


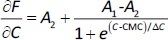

(1)

where *A*_1_ and *A*_2_ are the horizontal asymptotic values corresponding to the premicellar and postmicellar regions, respectively; CMC is the breakpoint of curve *F vs. C*; and Δ*C* is the range where the sudden fluorescence change occurs (the width of the transition between the premicellar and the postmicellar regions). Equation fitting to the data provides the CMC calculated value.
- Second derivative or Philips’ method (method 3). Here, data are fitted to the following Gaussian equation (Eq. 2) to calculate the CMC value.


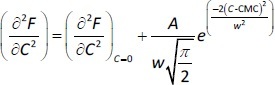

(2)

where (∂^2^*F*/∂*C*^2^)_C=0_ is the baseline offset, *A* is the area under the curve from the baseline and w is the width of the peak at half height.
- Carpena’s method [19] (method 4). In this method, *F vs. C* data are fitted to a sigmoidal model (Eq. 3) to obtain CMC values.


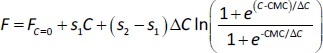

(3)

where *F*_C=0_ is the fluorescence measured before starting the NaTc additions, and *s*_1_ and *s*_2_ are the slopes of the premicellar and postmicellar linear regions, respectively.

## Results and discussion

### NaTc micellization evaluation: comparison of the methods for data treatment

As NaTc presents intrinsic fluorescence, we first considered performing the fluorescence monitoring of the aggregation experiments without using probes. In this way, any contribution of the probes to the micellization process can be avoided, and the interpretation of the obtained results is simplified.

**Figure 3.**
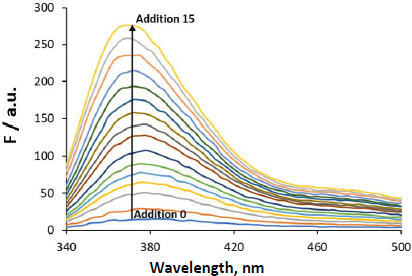
Fluorescence spectra recorded after successive additions of NaTc to a cuvette containing water

The experiments of NaTc micellization were first carried out using water as a medium. Several additions of the bile salt were made over the cuvette containing water, and the fluorescence spectra were recorded (Figure 3).

**Figure 4.**
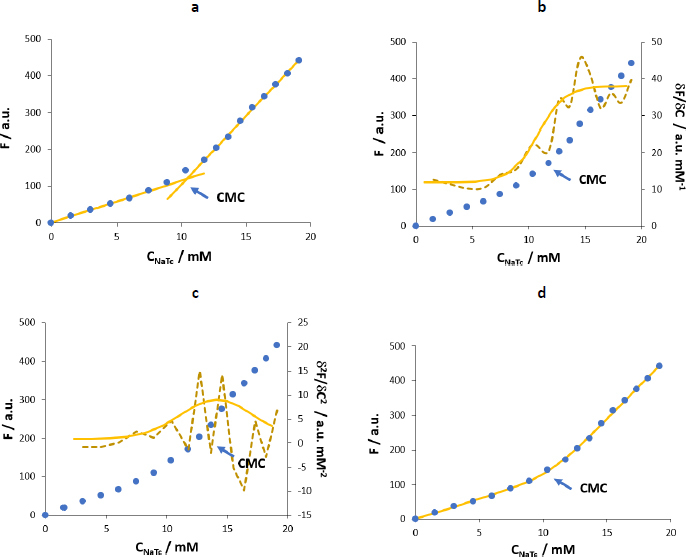
*F vs*. *C*_NaTc_ plots after successive additions of NaTc over a cuvette containing water at 25 °C. Experimental measurements are represented by the blue dots and the mathematical fitting by the yellow solid lines using a - method 1, b - method 2. The secondary axis corresponds to the first derivative (dashed line: first derivative calculated from experimental data); c - method 3. The secondary axis corresponds to the second derivative (diamonds: second derivative calculated from experimental data), d - method 4.

Next, data treatment was done using the four mathematical fitting methods described in the calculations subsection. Figure 4 shows the *F* (λ_ex_ = 310 nm, λ_em_ = 364 nm) *vs. C*_NaTc_ experimental plots and the mathematical fitting approaches for the same set of experiments.

The experimental points show that NaTc fluorescence intensity increases proportionally to the concentration of surfactant added (up to *C*_NaTc_ ~9 mM). At NaTc concentrations higher than about 11 mM, a linear trend also exists but with a higher slope. In the range 9-11 mM, a transition region is observed. The CMC and Δ*C* values calculated according to methods 1 to 4 are shown in Table 1.

**Table 1.** Aggregation parameters of NaTc in water obtained by monitoring its fluorescence at 25 °C and using four different calculation methods.

	CMC (SD*) / mM	ΔC (SD*) / mM
Method 1	10.4	-
Method 2	10.8 (0.06)	1.2 (0.6)
Method 3	14(1)	-
Method 4	10.6 (0.03)	1.1 (0.3)

*SD – standard deviation

The estimated CMC values by methods 1, 2 and 4 are equivalent. Method 1 is very useful for fast estimation of CMC value. However, the selection of the points for establishing the straight lines is to some extent subjective, and it does not provide a quantitative value for the range of micellization. In the case of method 2, although the sigmoidal curve provides equivalent CMC values compared to the ones obtained using other methods, the residuals (difference between the experimental and the calculated first derivative values) are high. This is especially relevant in the postmicellar zone, even though the fluorescence intensity variation in this region is important. As a result, the obtained CMC has a high uncertainty value, and for this reason the method was discarded in the following experiments. In the case of method 3, the estimated CMC value is 3 mM units higher than the other ones and, again, the residuals in the postmicellar region are high. Hence, it would not be suitable for the evaluation of CMC in the current experiments. Further, the model does not consider Δ*C*. Finally, the fit in method 4 provides a coherent CMC value with low residuals. Moreover, it allows calculating the range where the major micellization process occurs, Δ*C* = 1.1 (0.3) mM. In fact, this is the method recommended by Goronja *et al*. [16] when the transition between the premicellar and the postmicellar regions is not sharp but shows a gradual curvature, as in the case of NaTc.

After the evaluation of four different fitting methodologies, we conclude that the first and fourth methods are the ones more suitable for the determination of the CMC of NaTc. After evaluating all data, we can conclude that Carpenas’s method provides good fits and allows an estimation of the micellization range in addition to the CMC value. The first method is fast and simple. Although it does not provide information about the micellization range, we also consider it for the CMC value determination. Moreover, the obtained value can be used as a starting point for the iterative processes required in Carpena’s method.

### NaTc micellization evaluation: fluorophore suitability depending on the medium

According to the literature, the micellization of NaTc can be evaluated using different probes [7,11]. In the present work, we discarded probes with large molecular sizes to avoid the presence of probe aggregates that may make the interpretation of the results difficult. As a result, we first selected naphthalene and pyrene, aromatic nonpolar probes of small size that were soluble in different bile salts at millimolar levels [20]. Additionally, we selected two weak bases ionized at the pH of work (propranolol and tetracaine) that may be able to interact with both the hydrophobic and the hydrophilic regions of the NaTc micelles and, hence, would present different fluorescence when they are free and hosted on the micelle.

**Figure 5.**
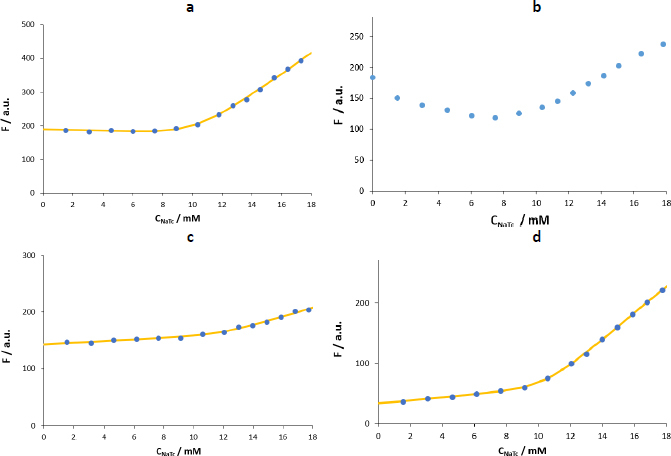
*F vs*. *C*_NaTc_ plots in water at 25 °C using as fluorophore: a - naphthalene, b - pyrene, c – propranolol, d - tetracaine. Experimental measurements are represented by blue dots and the fitting obtained through Carpena’s method by the solid yellow line.

We determined the CMC of NaTc by monitoring the change in fluorescence intensity of the different probes and also from NaTc itself in two different media (water and a maleic buffer that mimics the fasted state of the intestine fluids). Figure 5 shows the *F vs. C*_NaTc_ plots obtained using the four probes and water as a medium.

Naphthalene, propranolol and tetracaine show linear *F vs*. *C*_NaTc_ behaviour in the premicellar and postmicellar zones but with a higher slope in the second case. Also, a transition zone is observed.

In the case of pyrene (Figure 5b), the fluorescence intensity decreases upon the first additions of NaTc. This phenomenon was already observed by other authors using this same probe and may be due to the formation of pyrene-NaTc premicellar aggregates [12,17]. This association would involve non-fluorescent species that would lead to the fluorescence quenching observed during the first additions of the surfactant to the cuvette containing the fluorophore [21], until pyrene is effectively incorporated into the NaTc micelle. In the present study, we have used very low amounts of pyrene (0.03 μM) to disfavour the premicellar aggregation equilibrium, but the quenching effect is still appreciable. Therefore, the modelling does not provide accurate results in the determination of micellization parameters. Further, when we reduced the amounts of pyrene, we could not use the pyrene 1:3 ratio method. This method monitors the fluorescence intensity of pyrene at λ_exc_ = 335 nm and λ_emi_ = 373 nm (1^st^ vibronic band) and λ_emi_ = 384 nm (3^rd^ vibronic band). However, the third vibronic band is not well defined at 0.03 M and, therefore, cannot be accurately monitored.

Hence, we have discarded pyrene in favour of other probes that allow the evaluation of micellization events with higher selectivity and sensitivity and using simple mathematical modelling.

Table 2 shows the results obtained for the three remaining probes and NaTc itself using method 1 and method 4 to fit the data.

**Table 2.** Aggregation parameters of NaTc in water at 25 °C and monitoring different fluorophores

	Method 1	Method 4	
	CMC, mM	CMC, mM	Δ*C* / mM
NaTc	10.5 (0.6)	10.6 (0.7)	1.0 (0.3)
Naphthalene	10.4 (0.4)	10.7 (0.6)	0.8 (0.2)
Propranolol	10.7 (0.5)	10.9 (0.8)	0.8 (0.2)
Tetracaine	10.1 (0.3)	10.2 (0.2)	1.2 (0.3)

The standard deviation is shown in parentheses

Table 2 shows the results obtained for the three remaining probes and NaTc itself using method 1 and method 4 to fit the data.

Here, there is a good agreement between the CMC values obtained from the two different calculation methods. Further, the direct monitoring of NaTc fluorescence or that of probes soluble in NaTc micelles does not show significant differences in the aggregation parameters (CMC and Δ*C*), and hence the use of external probes could be avoided. The average CMC calculated value (10.5 (0.6) mM) is in the range of the ones reported using fluorescence at 20-25 °C and different probes (8-12 mM) [7,11] and slightly lower than the value obtained at 35 °C (15 mM) [12].

**Figure 6.**
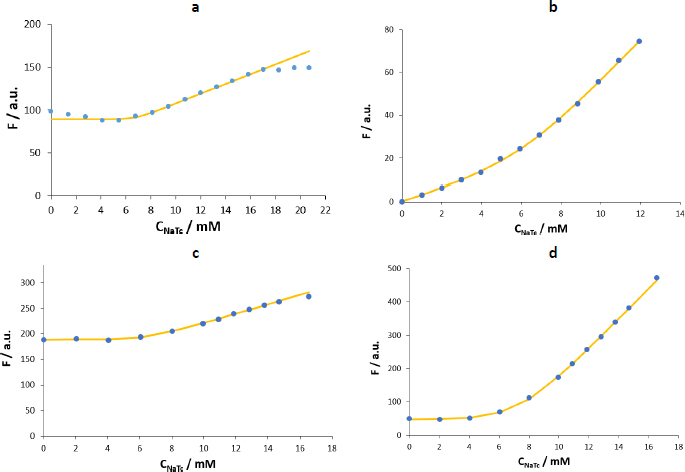
*F vs*. *C*_NaTc_ plots in maleic buffer at 25 °C using as fluorophore: a – NaTc, b – naphthalene, c **–** propranolol, d - tetracaine. Experimental measurements are represented by blue dots and the fitting obtained through Carpena’s method by the solid yellow line.

Figure 6 shows the *F vs*. *C*_NaTc_ plots obtained in maleic buffer (pH 6.5, *I* = 120 mM). The first difference observed in maleic buffer compared to water is that when the fluorescence of the NaTc is monitored (Figure 6a), its intensity is 2-3 times lower, and the dispersion of the results between replicates is high (Table 3). Therefore, using NaTc as an automarker is not recommended under these experimental conditions.

**Table 3.** Aggregation parameters of NaTc in maleic buffer (pH 6.5, *I* = 120 mM) at 25 °C and monitoring different fluorophores. In parentheses the standard deviation.

	Method 1	Method 4	
	CMC, mM	CMC, mM	Δ*C* / mM
NaTc	6.4 (0.9)	6.1 (1.4)	1.2 (1.2)
Naphthalene	7.0 (0.4)	6.9 (0.6)	0.6 (0.2)
Propranolol	6.9 (0.3)	7.1 (0.6)	0.8 (0.2)
Tetracaine	6.9 (0.4)	7.1 (0.4)	1.1 (0.3)

In the case of naphthalene, the fluorescence intensity increase after the formation of the micelles is lower than when working with water. Moreover, as observed previously for pyrene, the fluorescence is quenched during the additions previous to the formation of the micelles (Figure 6b). This behaviour differs from the one using the same probe in water (Figure 5a). Naphthalene has a rigid planar structure, like pyrene, but is half-sized. Hence, in water, naphthalene would be stable enough to avoid the formation of aggregates at low concentrations of the biosurfactant. However, when using the maleic buffer with much higher ion strength (*I* = 120 mM), the stability would be compromised, and aggregates similar to those formed by pyrene in water are present in the system. In this work, this effect has been partially mitigated by using low amounts of the probe (2 M) so that the fits obtained using naphthalene as a probe have been considered for the CMC calculation. However, the use of propranolol or tetracaine is advised in this case.

The shape of the plots for propranolol and tetracaine is similar in maleic buffer and water but with an inflection point at lower concentrations of added surfactant (6-8 mM) and with higher fluorescence intensity increase in the postmicellar zone. Among the two polar probes, tetracaine presents a fluorescence enhancement higher than that of propranolol (Figure 6 c and d) and is more sensitive and precise (as shown in Table 3, the relative standard deviation is lower). Furthermore, previously reported values on the micellization of NaTc in water show that the CMC value is in the range of 8-12 mM (20-25 °C, using fluorescence and NMR measurements) [7,11,15]. When using osmometry, the reported CMC values at 20 and 30 °C are lower (~6.5 mM), probably because this complementary technique can detect a physical change related to the formation of aggregates before the other techniques do.

Table 3 shows the corresponding results obtained using the two selected fitting methods. CMC values in the biorelevant buffer (7.0 (0.5) mM) are lower than those in water (10.5 (0.6) mM), and differences in the aggregation range (Δ*C* ~0.9 mM) are not significant. The high number of ions in the medium would decrease the repulsions between the polar regions of NaTc, and also the exposure of the nonpolar groups to the surrounding water. In consequence, the NaTc micellization would be promoted. This phenomenon was observed when experiments were performed in media (water and acetate buffer) with growing amounts of sodium chloride [12,22] and other surfactants [23].

Despite the good agreement in the results obtained with the three probes, in the case of naphthalene, the concentration of the probe should be carefully defined to avoid the formation of non-desired aggregates in the initial measurements. The literature does not report studies working with similar experimental conditions. Studies under other buffering conditions but with similar ion strength and temperature indicate that the CMC of NaTc is 3-9 mM [22,24,25], in the same order of magnitude as the values reported herein.

## Conclusions

In the present study, NaTc micellization has been evaluated by fluorescence using different approaches. First, experiments were performed in water to evaluate different data treatment procedures to evaluate the CMC. In the case of NaTc, a surfactant that aggregates gradually, Carpena’s method is the most appropriate as it provides the most accurate CMC and Δ*C* values. Monitoring the fluorescence of different fluorophores (NaTc itself and the probes naphthalene, propranolol and tetracaine) has provided equivalent results. However, pyrene would form aggregates that would avoid the accurate determination of the CMC. When a medium that mimics the pH and the ion strength of fasted-state intestinal fluids is used, NaTc fluorescence is not high enough, and the precision of the measurements is compromised; hence, the use of probes is advised.

The comparison of the CMC values in water (10.5(0.6) mM) and in maleic buffer (7.0(0.5) mM) reveals that the biorelevant conditions favours NaTc micellization, which in turn may allow faster solubilization of ingested lipids, vitamins, or low-polar drugs.

## References

[1] Jafari M. Mehrnejad F. Rahimi F. Asghari S.M., The molecular basis of the sodium dodecyl sulfate effect on human ubiquitin structure: A molecular dynamics simulation study, Scientific Reports 8 (2018) 2150. https://doi.org/10.1038/s41598-018-20669-7 10.1038/s41598-018-20669-7 29391595 PMC5794983

[2] Malik N.A., Solubilization and interaction studies of bile salts with surfactants and drugs: a review, Applied Biochemistry and Biotechnology 179 (2016) 179-201. https://doi.org/10.1007/s12010-016-1987-x 10.1007/s12010-016-1987-x 26781714

[3] Parekh P.Y. Patel V.I. Khimani M.R. Bahadur P., Self-assembly of bile salts and their mixed aggregates as building blocks for smart aggregates, Advances in Colloid and Interface Science 312 (2023) 102846. https://doi.org/10.1016/j.cis.2023.102846 10.1016/j.cis.2023.102846 36736167

[4] Matsuoka K. Yamamoto A., Study on micelle formation of bile salt using nuclear magnetic resonance spectroscopy, Journal of Oleo Science 66 (2017) 1129-1137. https://doi.org/10.5650/jos.ess17063. 10.5650/jos.ess17063 28966306

[5] Carey M.C. Small D.M., Micelle Formation by Bile Salts Physical-Chemical and Thermodynamic Considerations, Archives in Internal Medicine 130 (1972) 502-527. https://doi.org/10.1001/archinte.1972.03650040040005 10.1001/archinte.1972.03650040040005 4562149

[6] Matsuoka K. Moroi Y., Micelle formation of sodium deoxycholate and sodium ursodeoxycholate (Part 1), Biochimica Biophysica Acta 1580 (2002) 189-199. www.bba-direct.com 10.1016/s1388-1981(01)00203-7 11880243

[7] Meyerhoffer S.M. McGown L.B., Critical micelle concentration behavior of sodium taurocholate in water, Langmuir 6 (1990) 187-191. https://doi.org/10.1021/la00091a030 10.1021/la00091a030

[8] Roda A. Hofmann A.F. Mysels K.J., The influence of bile salt structure on self-association in aqueous solutions, Journal of Biological Chemistry 258 (1983) 6362-6370. https://www.jbc.org/article/S0021-9258(18)32418-9/pdf 6853487

[9] Rangel-Yagui C. de O. Pessoa A. Tavares L.C., Micellar solubilization of drugs, Journal of Pharmacy and Pharmaceutical Sciences 8 (2005) 147-163. https://sites.ualberta.ca/~csps/JPPS8(2)/C.Rangel-Yagui/solubilization.pdf 16124926

[10] Mukherjee B. Dar A.A. Bhat P.A. Moulik S.P. Das A.R., Micellization and adsorption behaviour of bile salt systems, RSC Advances 6 (2016) 1769-1781. https://doi.org/10.1039/c5ra20909a 10.1039/c5ra20909a

[11] Li G. McGown L.B., Model for bile salt micellization and solubilization from studies of a “polydisperse” array of fluorescent probes and molecular modeling, Journal of Physical Chemistry 98 (1994) 13711-13719. https://doi.org/10.1021/j100102a043 10.1021/j100102a043

[12] Matsuoka K. Maeda M. Moroi Y., Micelle formation of sodium glyco- and taurocholates and sodium glyco- and taurodeoxycholates and solubilization of cholesterol into their micelles, Colloids Surfaces B 32 (2003) 87-95. https://doi.org/10.1016/S0927-7765(03)00148-6 10.1016/S0927-7765(03)00148-6

[13] Pigliacelli C. Belton P. Wilde P. Qi S., Probing the molecular interactions between pharmaceutical polymeric carriers and bile salts in simulated gastrointestinal fluids using NMR spectroscopy, Journal of Colloid Interface Science 551 (2019) 147-154. https://doi.org/10.1016/j.jcis.2019.05.002 10.1016/j.jcis.2019.05.002 31075629

[14] Ravidiandjran G., Acoustical studies on the effect of urea on the micelles of aqueous surfactants, Indian Journal of Physics 76B (2002) 277-282. https://azpdf.net/document/y4wx85mr-acoustical-studies-effect-urea-micelles-aqueous-surfactants.html

[15] Funasaki N. Fukuba M. Kitagawa T. Nomura M. Ishikawa S. Hirota S. Neya S., Two-dimensional NMR study on the structures of micelles of sodium taurocholate, Journal of Physical Chemistry B 108 (2004) 438-443. https://doi.org/10.1021/jp030899h 10.1021/jp030899h 16852187

[16] Goronja J.M. Ležaić A.M.J. Dimitrijević B.M. Malenović A.M. Stanisavljev D.R. Pejić N.D., Determination of critical micelle concentration of cetyltrimethyl- ammonium bromide: Different procedures for analysis of experimental data, Hemijska Industrija 70 (2016) 485-492. https://doi.org/10.2298/HEMIND150622055G 10.2298/HEMIND150622055G

[17] Aguiar J. Carpena P. Molina-Bolívar J.A. Carnero Ruiz C., On the determination of the critical micelle concentration by the pyrene 1:3 ratio method, Journal of Colloid Interface Science 258 (2003) 116-122. https://doi.org/10.1016/S0021-9797(02)00082-6 10.1016/S0021-9797(02)00082-6

[18] Biorelevant, Biorelevant Media, (2024). https://biorelevant.com/ (accessed March 3, 2024).

[19] Carpena P. Aguiar J. Bernaola-Galván P. Carnero Ruiz C., Problems associated with the treatment of conductivity-concentration data in surfactant solutions: Simulations and experiments, Langmuir 18 (2002) 6054-6058. https://doi.org/10.1021/la025770y 10.1021/la025770y

[20] Ninomiya R. Matsuoka K. Moroi Y., Micelle formation of sodium chenodeoxycholate and solubilization into the micelles: Comparison with other unconjugated bile salts, Biochimica e Biophysica Acta – Molecular and Cell Biology of Lipids 1634 (2003) 116-125. https://doi.org/10.1016/j.bbalip.2003.09.003 10.1016/j.bbalip.2003.09.003 14643799

[21] Piñeiro L. Novo M. Al-Soufi W., Fluorescence emission of pyrene in surfactant solutions, Advances in Colloid Interface Science 215 (2015) 1-12. https://doi.org/10.1016/j.cis.2014.10.010 10.1016/j.cis.2014.10.010 25466688

[22] Thongngam M. McClements D.J., Isothermal titration calorimetry study of the interactions between chitosan and a bile salt (sodium taurocholate), Food Hydrocolloids 19 (2005) 813-819. https://doi.org/10.1016/j.foodhyd.2004.11.001 10.1016/j.foodhyd.2004.11.001

[23] Fuguet E. Ràfols C. Rosés M. Bosch E., Critical micelle concentration of surfactants in aqueous buffered and unbuffered systems, Analytica Chimica Acta 548 (2005) 95-100. https://doi.org/10.1016/j.aca.2005.05.069 10.1016/j.aca.2005.05.069

[24] Grijalva-Bustamante G.A. Quevedo-Robles R. V. del Castillo-Castro T. Castillo-Ortega M.M. Encinas J.C. Rodríguez-Félix D.E. Lara-Ceniceros T.E. Fernández-Quiroz D. Lizardi-Mendoza J. Armenta-Villegas L., A novel bile salt-assisted synthesis of colloidal polypyrrole nanoparticles, Colloids and Surfaces A 600 (2020) 124961. https://doi.org/10.1016/j.colsurfa.2020.124961 10.1016/j.colsurfa.2020.124961

[25] de Oliveira C. Khatua B. El-Kurdi B. Patel K. Mishra V. Navina S. Grim B.J. Gupta S. Belohlavek M. Cherry B. Yarger J. Green M.D. Singh V.P., Thermodynamic interference with bile acid demicelleization reduces systemic entry and injury during cholestasis, Scientific Reports 10 (2020) 8462. https://doi.org/10.1038/s41598-020-65451-w 10.1038/s41598-020-65451-w 32439972 PMC7242474

